# LENS: web-based lens for enrichment and network studies of human proteins

**DOI:** 10.1186/1755-8794-8-S4-S2

**Published:** 2015-12-09

**Authors:** Adam Handen, Madhavi K Ganapthiraju

**Affiliations:** 1 Department of Biomedical Informatics, University of Pittsburgh, Pittsburgh, PA, 15206, USA

## Abstract

**Background:**

Network analysis is a common approach for the study of genetic view of diseases and biological pathways. Typically, when a set of genes are identified to be of interest in relation to a disease, say through a genome wide association study (GWAS) or a different gene expression study, these genes are typically analyzed in the context of their protein-protein interaction (PPI) networks. Further analysis is carried out to compute the enrichment of known pathways and disease-associations in the network. Having tools for such analysis at the fingertips of biologists without the requirement for computer programming or curation of data would accelerate the characterization of genes of interest. Currently available tools do not integrate network and enrichment analysis and their visualizations, and most of them present results in formats not most conducive to human cognition.

**Results:**

We developed the tool Lens for Enrichment and Network Studies of human proteins (LENS) that performs network and pathway and diseases enrichment analyses on genes of interest to users. The tool creates a visualization of the network, provides easy to read statistics on network connectivity, and displays Venn diagrams with statistical significance values of the network's association with drugs, diseases, pathways, and GWASs. We used the tool to analyze gene sets related to craniofacial development, autism, and schizophrenia.

**Conclusion:**

LENS is a web-based tool that does not require and download or plugins to use. The tool is free and does not require login for use, and is available at http://severus.dbmi.pitt.edu/LENS.

## Background

More and more studies are being carried out to identify genes that are associated with diseases or traits, enabled by advances in high-throughput technology - be it gene expression microarrays of last decade, or the whole exome or whole genome sequencing technologies of today. The next urgent question that needs to be answered is how these genes play a role in the said diseases or traits. Network analysis has been applied to discover the functional characteristics of these genes and to identify their role in the pathways associated with diseases. Such analyses were made possible by the availability of curated protein-protein interaction (PPI) databases such as STRING [1], MINT [2], BioGRID [3], HPRD [4] and DIP [5]. Network analysis using previously published PPI data has been carried out extensively, some examples of which are the study of craniofacial development [6], Hutchinson-Gilford progeria syndrome [7], glioblastoma [8] and general drug discovery [9]. Recognizing the capability of network based studies, there have been focused efforts to determine disease specific interactomes using high-throughput technology such as yeast 2-hybrid (Y2H) and to annotate proteins and even make inferences about functions and relations to new diseases. This concept has recently been applied to Huntington's disease [10], methyltransferase activity [11], and blood coagulation after Hepatitis E infection [12], among others.

There are a large number of online tools available freely for the analysis of PPIs. The common workflow for using PPIs to study diseases is to retrieve a sub-network of the genes from the interactome and then study whether the network is enriched for any pathways or biological functions. The aforementioned PPI databases all allow users to download the PPIs as a list, but network analysis usually requires some programming skills. There are web-based tools for gathering the gene set specific PPI networks to carry out some network analysis. PPISURV [13] looks across multiple PPI databases to retrieve interactions of queried genes. ContextNET [14] goes a step further to find not only immediate interactors, but also paths of PPIs to connect queried genes. These tools provide results in a tabular format which can be exported to other tools like DAVID [15], or Babelomics [16] to study pathway/biological term enrichment in the network. However, these tools lack graphical visualization of the output and do not present the information in a format that can be easily interpreted or assimilated by the users.

Other tools offer more, collecting PPIs, performing network analyses, and even offering some visual representation of the data that is more useful to researchers. PINA2 [17], DTOME [18], and Graphite Web [19] are tools that provide this sort of pipeline. All of these tools require the installation of additional software or plugins, like Java, Flash, or even Cytoscape [20].

We developed a web-based tool called LENS: *Lens for Enrichment and Network Studies of Proteins*, that takes a set of genes as input and with a single click of a button presents the results of a number of network-based analyses, including statistically enriched pathways, etc. in the interactome; the tool is freely available and requires no plugins or software downloads. In addition to reporting the significance of overlap of the network with various pathways and disease-associated genes, the structure of the network itself is also analyzed, comparing the connectivity of queried genes against random gene sets to help researchers make inferences of the potential significance of their results.

## Implementation

**Figure 1:**
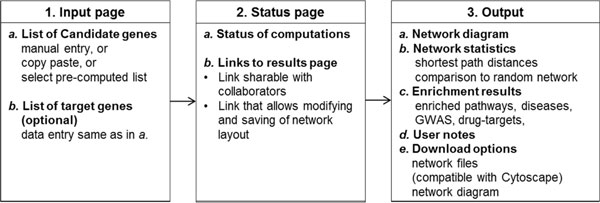
**Overview of the features of LENS**. LENS accepts lists(s) of genes in the input page and immediately shows the status page upon submission, which contains the links to the results page. Users may choose to wait for results to be computed, or save the links and return at a later time to view the results. They will be able to access the results by saving the URLs shown.

LENS is a multifaceted tool. An overview of LENS is shown in Figure 1. It accepts lists of genes as input, and outputs many types of results. The features implemented in the web application are described here.

### Data sources

The sources of the data behind LENS are as follows: PPIs are collected from HPRD and BioGRID databases. Binary biophysical PPIs are collected from HPRD by choosing so named file from the downloaded flat files. Similar PPIs are collected from BioGRID by selecting those interactions that have an MI code of 0417. Disease to gene mappings are obtained from KEGG [21], pathway to gene mappings are collected from REACTOME [22], drug to gene mappings are obtained by parsing DrugBank [23] data, and finally GWAS diseases/traits to genes mappings are collected from NHGRI's GWAS catalog [24].

### Backend

When the user submits their gene lists, the data is sent to our server and processed by several Python scripts which utilize the NetworkX library [25]. NetworkX is a library for graph/network construction and analysis. The data to populate the networks and perform enrichment is stored in a MySQL relational database, the same backend as Wiki-Pi. The results of the analyses are stored in JSON files on our servers so users can access results in the future as well. The status page users see while the results are being computed makes AJAX calls to our server to check for the presence of the output JSON files. When the user eventually moves on to the results page, the visualization for the data is an implementation of Data Drive Documents (D3), a JQuery library [26]. D3 is used for the network visualization and the Venn diagrams in the results page. Additional rendering of data, such as creating the individual buttons and the general layout of the website, is a combined effort of Bootstrap CSS and FuelPHP.

### Input to LENS

LENS accepts either one or two gene sets as input. The genes in the first set are called *candidate genes *and the genes in the optional second set are called *target genes*. Genes may be provided as HUGO approved symbols, or as Entrez or UniProt IDs. Users can type or paste a list of genes into the input box. When a user enters the gene list manually, LENS checks for the authenticity and availability of those genes in our database, prior to sending them to next page for further analysis. LENS also has pre-made lists of genes associated with known diseases, pathways, and GWASs (updated monthly) that the user may select from instead of typing in their own gene lists. When a user starts typing the name of a pathway or disease or drug into the appropriate input box, LENS suggests possible auto-complete options, from which the user can select an item of interest (say 'Vitamin D levels NHGRI GWAS'). The auto-complete options include NHGRI GWAS [27], REACTOME pathways [22], KEGG diseases [21] and DrugBank drugs [23], in that order. Selecting a specific option from the drop-down list automatically populates the associated genes into the input text box normally used for manual input.

### Status page

Upon submitting the job, LENS takes users to the next page which shows the status of computing the results. Two URLs are presented - one for "viewing and sharing" and another for "viewing and editing." While both links go to the results page, the latter allows users to save changes to the notes and title of the results. Users may share the links with their collaborators so that they can see the results of analysis of the input genes. These links may also be used in publishing the results as the page may be accessed by anyone with whom the link is shared. While the results are still being computed, these links will return users to the loading page. The same links go to the results page when computations are complete. Thus, users need not wait at the computer while the results are being computed - they can save the link and return later to view the results. However, note that for small sets of input genes, LENS completes the computations fairly quickly (within a minute or two).

### PPI network graph

At the very top of the results page is a network view of the input genes and the PPIs that connect them. This visualization does not require any plugins; it works with all modern web browsers and even functions on smart phones and tablet devices. The network is interactive and allows users to click and drag nodes around to customize the appearance of the interactome. Users can also zoom/scale the network, hide non-connecting nodes, and toggle the visibility of gene labels.

**Figure 2:**
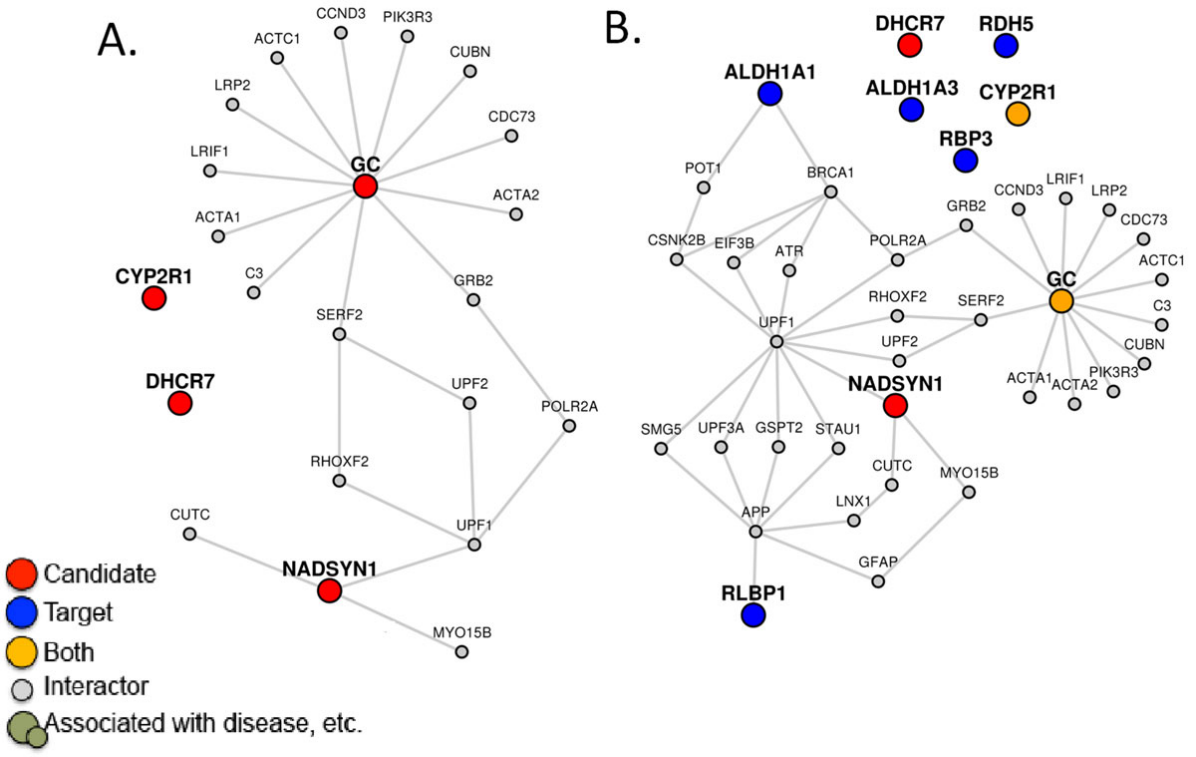
**Networks and statistics generated by LENS**. (A) A network generated with only candidate genes (shown in red), shows the immediate interactors and paths between them. (B) A network generated with candidate *and *target genes (shown in blue) shows the immediate interactors of candidate genes only, and the paths that connect the candidates to their closest targets. The network statistics the two networks are shown in Table 1.

LENS generates the network differently depending on whether one or two gene sets are given. The first gene set, called *candidate genes*, recruits all immediate interacting partners into the network. Figure 2A shows this first case, where candidate genes are shown as red colored nodes. If only candidate genes are given, LENS goes through each candidate gene and finds its nearest neighbor candidate gene in the interactome and includes the intermediate genes and interactions necessary to link them together (i.e. it includes the paths between them). If multiple nearest neighbors or multiple shortest paths to these neighbors are found, all of them are included. The second/optional gene set is called the *target genes*. Figure 2B shows the second case, where candidate genes are shown in red color, target genes are shown in blue color and genes common to both sets are shown in yellow. If target genes are given, LENS will find and include the shortest paths from each candidate gene to its closest neighbors among target genes instead of finding its neighbors among candidate genes. In this case, the network consists of direct interactors of candidate genes and the shortest paths from candidate to target genes. Immediate interactors of target genes are not included.

Every node and edge in the graph is also linked to Wiki-Pi [28]. Wiki-Pi is a web application that presents annotations of proteins in PPIs. LENS relies on Wiki-Pi for its data, including PPIs, pathways, diseases, drugs, and GWASs. When users follow nodes or edges to Wiki-Pi, they may also find links to protein structures, Gene Ontology terms, and tag clouds of common terms in publications.

### Network statistics

The connectivity in the sub-interactome network can be an indicator of how related the genes are. If the genes are highly connected it is likely that there is an association between them. Three values that describe the network connectivity are reported by LENS: minimum shortest path length, average shortest path length, and the count of disconnected nodes. Minimum shortest path length is the minimum of the lengths of shortest paths between every pair of candidate genes (or every pair of candidate and target genes). Average shortest path length, is the average of the lengths of shortest paths between every pair of candidate genes (or every pair of candidate and target genes). Multiple paths of equal length between a pair of nodes are not counted multiple times in computing the average. If there is no shortest path between the desired pair of nodes, their distance is set to be 17, which is 5 edges longer than the maximum length observed in the entire human PPIs network. This is done so as to give disconnected nodes a value for calculations of connectivity but keeping it significantly larger than the observed maximum distance; so we chose the number 17. Disconnected *target *genes are not counted, and do not affect the average. This also applies to the number of disconnected nodes reported. That is, only the number of disconnected candidate genes is given.

To put these numbers in perspective, corresponding numbers are reported for randomly generated networks. If only candidate genes were provided, five networks are generated using randomly selected genes in equal number to the original input size. The minimum shortest path length, average shortest path length, and number of disconnected nodes are averaged from the five networks and reported (see Table 1A). If target genes are given as input, three sets of random networks are generated (see Table 1B). The first selects random candidate genes as before, but retains the list of target genes. The second keeps the candidate genes from the input and randomizes the target genes. The last randomizes both gene sets.

**Table 1:** Network statistics when (A) only candidate genes are given and (B) when both candidate and target genes are given.

Network	Min. shortest path length	Avg. shortest path length	Disconnected nodes
***A. Network statistics corresponding to Figure 2A where only candidate genes are given***			

Candidate genes	4	14.83	2
Random genes	9	14.77	2.6

***B. Network statistics corresponding to Figure 2B where both candidate and target genes are given***			

Candidate to Target	3	10.25	2
Candidate to Random	8.4	12.9	2.8
Random to Target	11.2	14.15	3.2
Random to Random	8.8	13.5	3

### Enrichment statistics

The candidate genes are often those that are a result of a genome-wide association study or some other high-throughput data analysis study. As the outcomes of these studies are not limited by the knowledge in current literature about individual genes and proteins, several of these may be uncharacterized in terms of pathways, functions, drugs and diseases. Interactome analysis of these genes is typically carried out to study what pathways, diseases and biological functions are enriched in *their network*, so that their own pathways or functions may be hypothesized.

**Figure 3:**
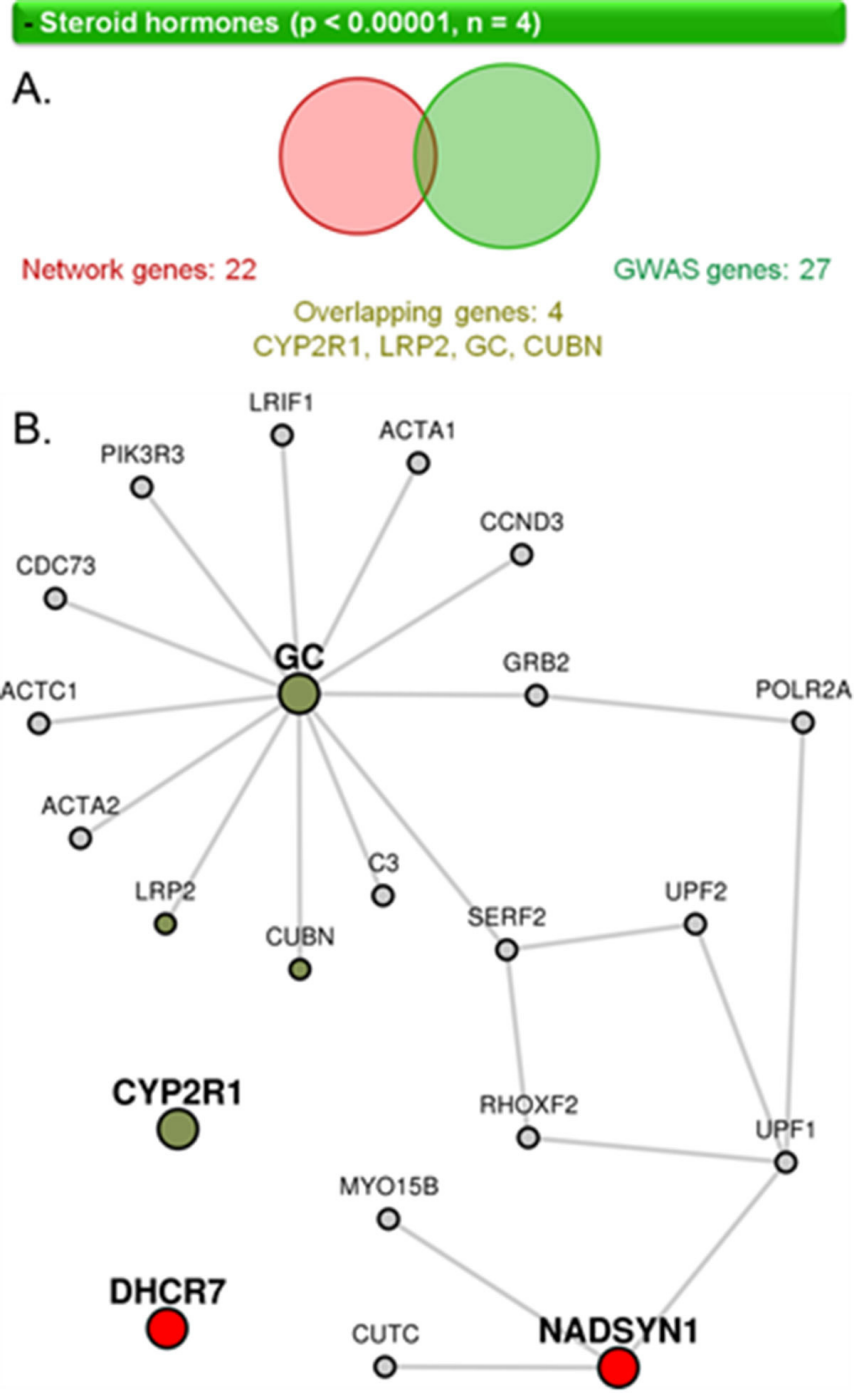
**Visualization of pathway enrichment shown by LENS**. (A) Venn diagram showing the overlap between candidate genes and a particular pathway, the steroid hormones pathway. The top horizontal bar shows the name of the pathway (or GWAS study or disease or drug name), the p-value of overlap and the number of genes that overlap between the two sets. In the Venn diagram also shows the total number of genes in the candidate set and the pathway, as well as the specific genes that overlap between the two. Each of the listed genes is linked to its corresponding page in Wiki-pi [28]. (B) When the horizontal bar corresponding to a feature (pathway, GWAS, etc.) is selected, its genes that overlap with the network are shown colored in a brownish-green color.

LENS computes the statistical significance of the presence of gene annotations in the network, namely pathways, diseases, drugs, and GWASs. For each annotation present in the network, LENS reports a p-value computed from Fisher's exact test, and depicts the overlap of genes in the network and genes with the annotation using a Venn diagram (Figure 3A). Genes with the annotation are also highlighted in the network view in green.

### Exporting the results

The network visual can be very useful for presenting results to others or as evidence in publications. The network as it appears can be downloaded in two formats, PNG and SVG. The image is stored at 600dpi resolution that would be of publication quality with even the smallest labels readable at this size. The SVG file is in XML format with only the node and edge coordinates included for users to add their own custom formatting of the network appearance.

A list of all genes in the network can be downloaded as a plain text file with either Entrez IDs, UniProt IDs, or HUGO symbols.

LENS data may also be downloaded in Cytoscape-compatible format: .SIF files of the entire network, or of just the shortest paths. These can be imported into Cytoscape for further viewing, formatting, annotating and analyses.

### Customizing

Downloading the graph is useful for figures or additional analysis, but showing the results of LENS as they appear on the website can be useful for demonstrating evidence to others. On the loading page of LENS, two URLs are provided, one of which allows customizing the output page to an extent. The editing URL allows users to change the title at the top of the page and add notes in the Notes tab at the bottom. After changes to these sections are made, clicking the "Save View" button will save the alterations on the server side. Then users returning to the page will see the changes to the title and notes. The other URL for "View and Share" allows users to see changes made, but will not let them alter the notes or title.

## Results and discussion

With more and more genome-wide associations studies being published in the recent years, there is growing interest in analyzing not only the functional associations of genes associated with a specific disease, but also how the different diseases relate to each other. This web application is an excellent resource for scientists who are interested in characterizing the genes in terms of their interactions and functions and the network in terms of the pathways and diseases enriched in it.

We demonstrate LENS through two case studies, one study pertaining to craniofacial development and another study pertaining to autism and schizophrenia.

### Craniofacial development replication study

**Figure 4:**
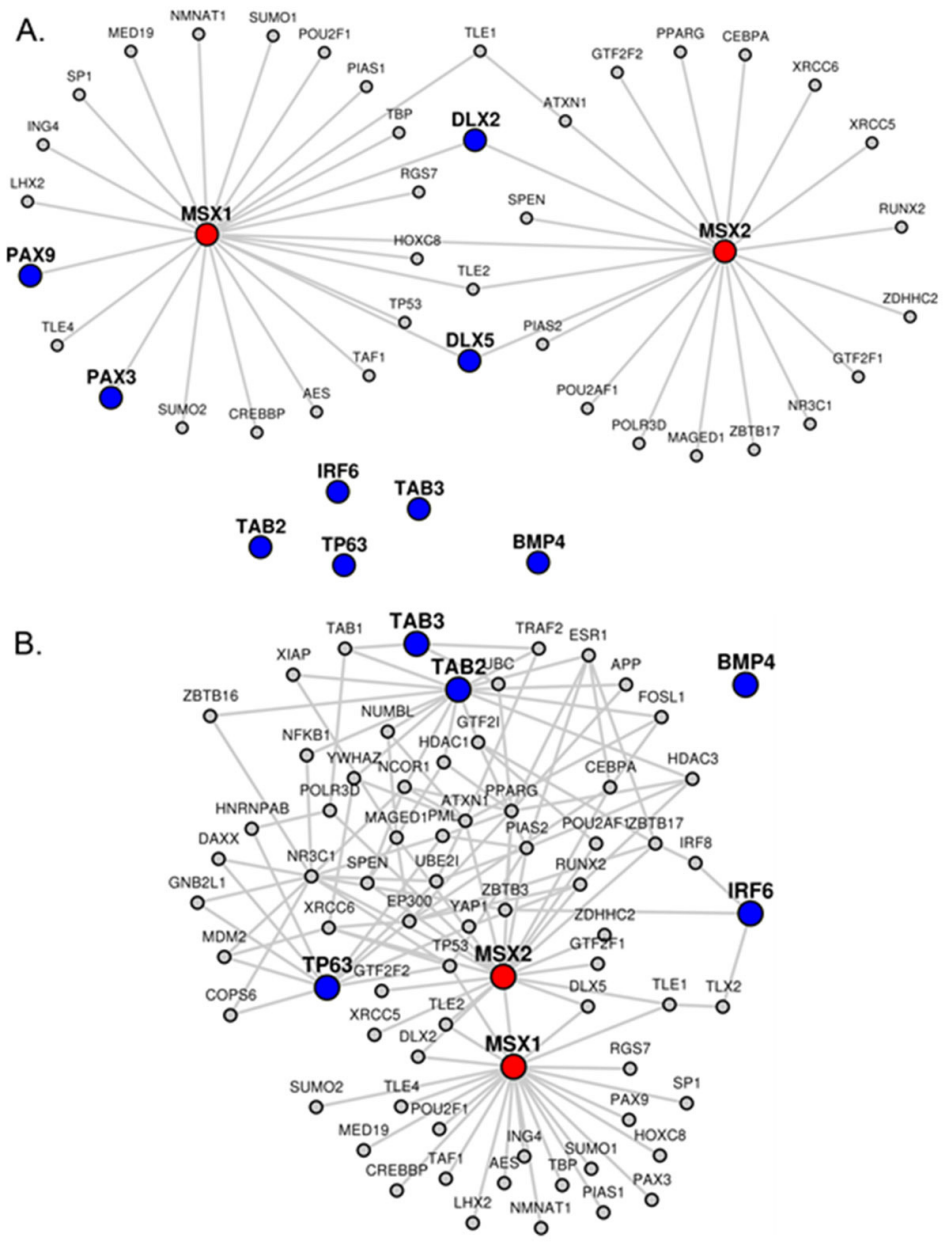
**Craniofacial study network analysis**. (A) Network corresponding to candidate genes MSX1 and MSX2 and target genes IRF6, TP63, DLX2, DLX5, PAX3, PAX9, BMP4, TAB2, and TAB3. (B) Network corresponding to candidate genes MSX1 and MSX2 and target genes IRF6, TP63, BMP4, TAB2, and TAB3.

In a previous study related to craniofacial development, network analysis of two genes MSX1 and MSX2 revealed several other genes of interest [6]. MSX1 and MSX2 were found to be related to IRF6, TP63, DLX2, DLX5, PAX3, PAX9, BMP4, TAB2, and TAB3. We used LENS with these genes and compared its performance with the tools used in their study (GeneDecks [29], DAVID [30,31], and STRING [1]). LENS shows a rather simple network when MSX1 and MSX2 were provided as candidate genes and the rest as target genes. When both candidate and target list are given, LENS attempts to find the shortest paths between each candidate gene and *its closest target *gene. In this example, no additional paths are added because MSX1 and MSX2 connected to four of the target genes directly through their PPIs (see Figure 4A for the network diagram and http://severus.dbmi.pitt.edu/LENS/index.php/results/view/53220bf5f158c/ for complete results including network connectivity). This does demonstrate though, that simply by providing MSX1 and MSX2 as input, PAX3, PAX9, DLX2, and DLX5 would all be identified as genes of interest. By removing these four genes from the target list and resubmitting the query, a very different network appears. The four genes are still found in this new network because they are interactors of MSX1 and MSX2, but as these genes are no longer target genes, LENS found new paths to connect MSX1 and MSX2 to a different closest target gene (see Figure 4B for the network diagram and http://severus.dbmi.pitt.edu/LENS/index.php/results/view/52dfbe4483da1/ for complete results). In the online results, one can see that the network statistics of the new network suggests relatedness between the candidate and target genes. The candidate genes are much more closely connected to target genes (average shortest path distance 2.5) than to same number of random genes (average shortest path distance 5.9). So also, the target genes are closer to candidate genes as compared to same number of random genes (average shortest path distance 15.6). Random computations are averaged over 5 separate runs.

Besides the network statistics, LENS also computed statistically enriched drugs, diseases, pathways, and GWASs in the network. Some of these are interesting to note given the hypothesis in the original publication, namely, that other genes that cross link with MSX1 and MSX2 are involved in some way with craniofacial deformities: two GWASs for cleft lip and facial morphology were found in the network, specifically for IRF6 and PAX3. The KEGG diseases also have interesting overlaps with several conditions related to facial morphology. PAX3 is picked out again for its relation with craniofacial-deafness-syndrome, and MSX2 was found in both enlarged parietal foramina/cranium bifidum and craniosynostosis. Relating to the original research, which had placed a particular emphasis on cleft palate and cleft lip, MSX2 was found in a specific syndrome for cleft palate (EEC syndrome), and three genes were all related to the original result - cleft lip with or without clef palate - with very high significance (p < 0.00001). What is also interesting is that MSX1, MSX2, IRF6, and PAX3 all appear to be connected through two other genes: TLX2 and TLE1. Because these genes seem to be the shortest common pathway between all of the proteins related to craniofacial development, it is possible that TLX2 and TLE1 are also involved in the process. Upon exploring the literature, we found that there is a link between TLX2 and the MSX genes as bone morphogenic proteins [32]. From wiki-pi [28], our website that shows comprehensive annotations of proteins, we find that TLE1 is not only known to be associated to *organ morphogenesis *Gene Ontology (GO) biological process term, but also has *anatomical structure morphogenesis *and *tissue morphogenesis *among GO terms enriched through its own interacting partners (see "GO annotations" tab and "Enriched GO terms among interacting partners" tab in http://severus.dbmi.pitt.edu/wiki-pi/index.php/gene/view/7088).

### Autism and schizophrenia

**Figure 5:**
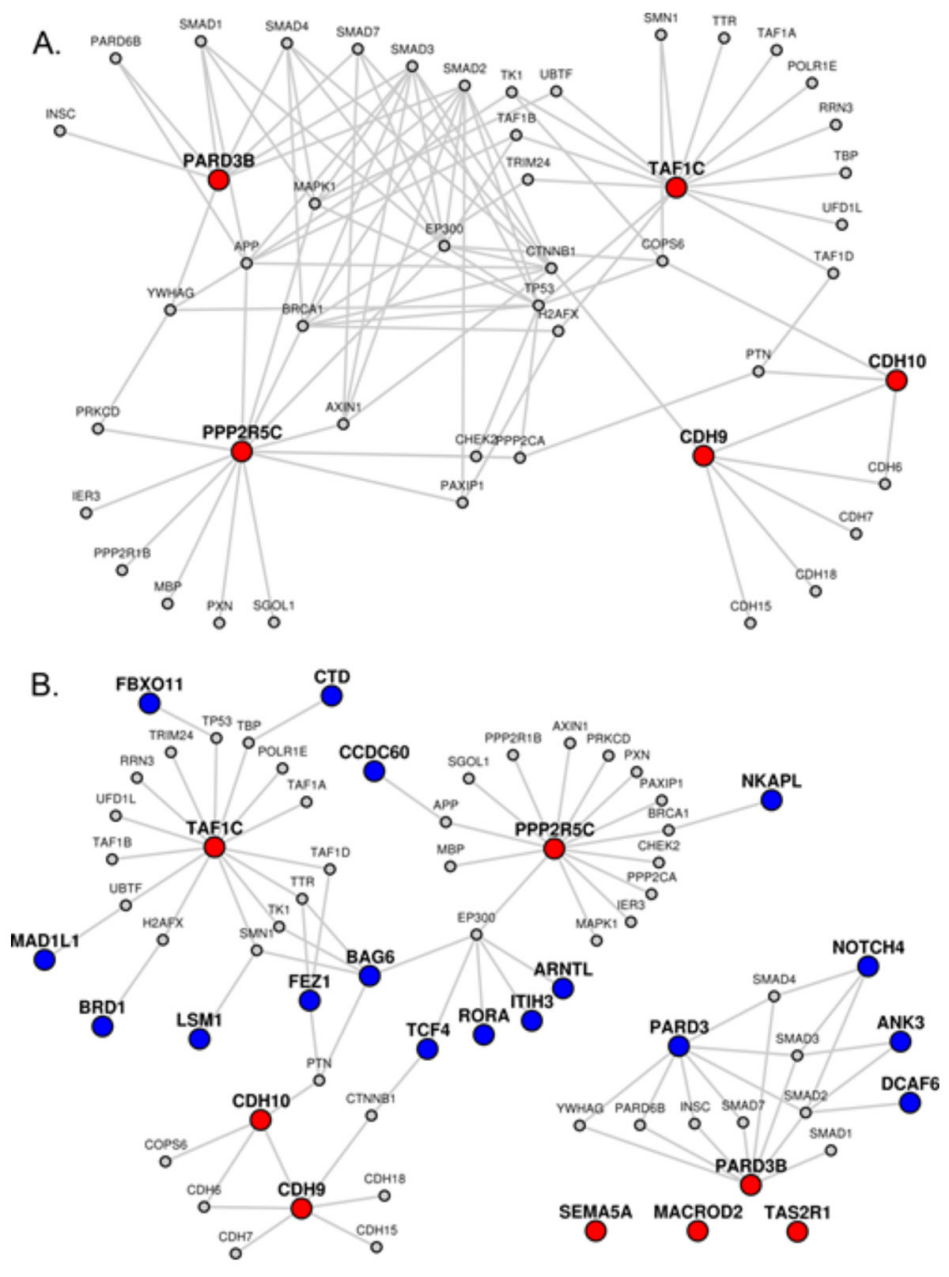
**Autism to Schizophrenia**. (A) Network generated with autism genes (input to LENS given using right side autocomplete list corresponding to ***autism NHRGI GWAS***) provided as candidates. (B) Network generated with autism genes (NHGRI GWAS) as candidates and schizophrenia genes (NHGRI GWAS) provided as targets.

Recent studies have shown that autism and schizophrenia are strongly interconnected; some genetic markers [33,34] and environmental conditions [35] have been identified to be potential common risk factors between the two conditions. We used LENS to see if the tool could find anything interesting to reveal the interrelation. The interactome of autism was constructed using the cumulated GWASs for the trait (see Figure 5A for the network diagram and http://severus.dbmi.pitt.edu/LENS/index.php/results/view/532b0e1b81054 for complete results including network connectivity). The result is a fairly crowded network, largely due to highly interactive proteins being found in the shortest paths, specifically TP53, EP300, APP, and MAPK1. The network enrichment shows some overlaps between inflammatory diseases (inflammatory bowel disease, Crohn's disease, and antiphospholipid syndrome), something of interest given the relation between mental disorders and inflammation [24]. Bipolar disorder also comes up as just barely significant (p = 0.0496). The autism network is also very well joined, judging from the network statistics. The shortest paths between nodes are much smaller for autism than for random networks.

We then took the same input of candidate genes and supplied schizophrenia associated genes from GWASs as target genes. See Figure 5B for the network diagram and http://severus.dbmi.pitt.edu/LENS/index.php/results/view/532b0e2762c8f/ for complete results. The network shows that autism associated genes are very closely connected to the schizophrenia genes. The network statistics show that autism relates strongly to most random gene sets also, which is likely explained by having hubs like EP300 and APP as immediate interactors.

**Figure 6:**
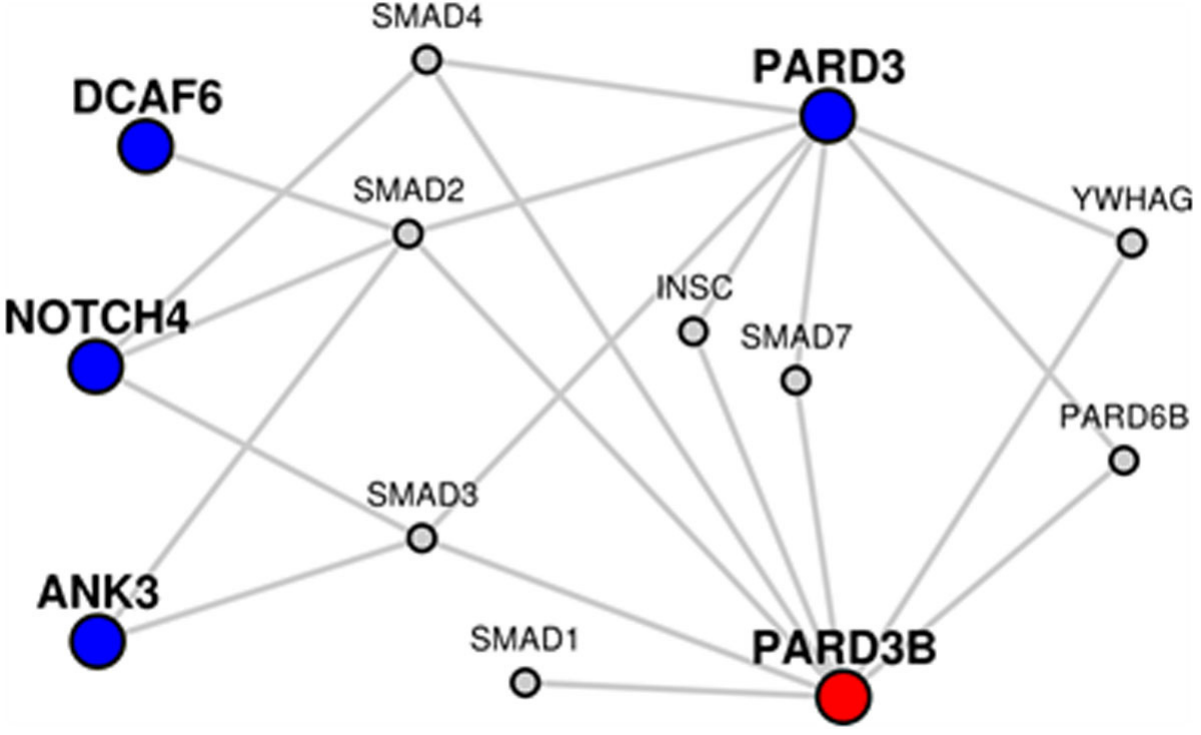
**Network and Enrichment analysis of a sub-network**. A sub-network from Figure 5 is resubmitted to LENS so that the pathways and other feature enriched in it may be studied.

Numbers aside, the shape of the autism-schizophrenia network shows an isolated module made from PARD3B to several schizophrenia genes. This module includes the SMAD genes, known to be associated with autism [36]. Of particular interest is the PARD6B gene. This gene is associated with bipolar disorder, and directly connects two genes related to autism and schizophrenia. The three genes, PARD3B, PARD6B, and PARD3, may play more of a role in mental disorder than the literature currently documents. We created a new network for just the module created from PAD3B to schizophrenia genes (see Figure 6 for the network diagram and http://severus.dbmi.pitt.edu/LENS/index.php/results/view/532b11b8eca85 for complete results). Enrichment studies of this module highlight many of the same enrichments found in the previous network, including bipolar disorder and the inflammatory diseases.

In the larger network of autism and schizophrenia genes, bipolar disorder and depression also appear to be much more significant traits with p-values less than 0.001. The immune/inflammatory diseases also show significance on the autism-schizophrenia network. Among the new diseases are asthma and several listings for immune responses to various infections.

### Comparisons to other tools

As mentioned earlier, there are numerous tools available for network or enrichment analyses. The major contributions of our tool is to offer both network and enrichment analysis in one tool, and to make that tool easy to use, without requiring users to download or install additional software or plugins. We compared LENS's functionality to that of three similar tools: VisAnt [37], UniHI [38], and GeneMANIA [39]. The details of the comparison may be seen in Table 2. While each tool has its own use and domain, LENS is the only one of the three that does not require any additional software to use.

**Table 2:** Comparison of LENS with other available tools.

Tool	Required software	Network analyses	Enrichments	Suggested Use
LENS	(None)	Shortest paths	Diseases, drugs, pathways, GWASs	Examine relatedness of genes and gene sets

VisANT	Yes (Java)	Degree distribution	Gene Ontology	Examining networks at different resolutions (e.g. PPI networks and disease networks)

UniHI	Yes (Flash)	Edge filters-^1^	Gene Ontology, diseases	Generating and filter networks from different sources of PPIs

GeneMANIA	Yes (Flash)	(None)	Gene Ontology	Enrichment and prioritization of genes by their Gene Ontology terms

^1^UniHI can selectively display edges based on various criteria

## Conclusions

LENS is an easy to use tool that computes a large number and enrichment statistics with a single click, and is entirely web-based with no requirement for or plugins or other software downloads. The results are easy to read and interpret, allowing researchers to draw conclusions or identify new genes of interest for further investigation. The results can be shared among collaborators via a web address. The nodes and edges are linked to the pages on Wiki-Pi that shows comprehensive annotations of corresponding genes and PPIs.

The ability to save the layout of nodes in the graph displayed on the results page will be developed soon, as well as Gene Ontology term enrichment analysis [40]. The objective of this work is to make the information and analyses tools available at the fingertips of biologists. With this objective, we will continue to add further features to the website. To gather input from the users about the most desired features to be added to LENS, we provide a community input page on the website where users may submit requests of additional features to be developed on LENS.

## Availability

**Project name: **Lens for the enrichment and network studies of proteins (LENS)

**Project home page: **http://severus.dbmi.pitt.edu/LENS

**Operating system(s): **Platform independent

**Programming language: **Perl, PHP, Python, and JavaScript

**Other requirements: **None

**License: **GNU

**Any restrictions to use by non-academics: **None

## Competing interests

Authors declare that they do not have competing interests.

## Authors' contributions

AH developed the network analysis and enrichment programs, built the web interface, carried out the use cases, and drafted the manuscript.

MKG conceived of the study, aided in the design of the web interface, and edited the manuscript.

## Declarations

The publication charges for this article were funded by NIMH/NIH grant 1R01MH094564.

This article has been published as part of *BMC Medical Genomics *Volume 8 Supplement 4, 2015: Joint 26th Genome Informatics Workshop and 14th International Conference on Bioinformatics: Medical genomics. The full contents of the supplement are available online at http://www.biomedcentral.com/bmcmedgenomics/supplements/8/S4.
